# Targeted Next-Generation Sequencing Identified Novel Compound Heterozygous Variants in the *CDH23* Gene Causing Usher Syndrome Type ID in a Chinese Patient

**DOI:** 10.3389/fgene.2020.00422

**Published:** 2020-04-30

**Authors:** Lianmei Zhang, Jingliang Cheng, Qi Zhou, Md. Asaduzzaman Khan, Jiewen Fu, Chengxia Duan, Suan Sun, Hongbin Lv, Junjiang Fu

**Affiliations:** ^1^Department of Pathology, The Affiliated Huaian No. 1 People’s Hospital of Nanjing Medical University, Huai’an, China; ^2^Key Laboratory of Epigenetics and Oncology, The Research Center for Preclinical Medicine, Southwest Medical University, Luzhou, China; ^3^Department of Ophthalmology, The Affiliated Hospital of Southwest Medical University, Luzhou, China

**Keywords:** Usher syndrome type ID, *CDH23* gene, missense mutation, targeted next-generation sequencing, genotype/phenotype correlation

## Abstract

Usher syndrome includes a group of genetically and clinically heterogeneous autosomal recessive diseases, such as retinitis pigmentosa (RP) and sensorineural hearing loss. Usher syndrome type I (USHI) is characterized by profound hearing impairment beginning at birth, vestibular dysfunction, and unintelligible speech in addition to RP. The relationships between the Usher syndrome causing genes and the resultant phenotypes of Usher syndrome have not yet been fully elucidated. In the present study, we recruited a Chinese family with Usher syndrome and conducted paneled next-generation sequencing, Sanger sequencing, segregation analysis, and expression profile analysis. The functional effects of the identified cadherin-related 23 (CDH23) pathogenic variants were analyzed. The M101 pedigree consisted of a proband and seven family members, and the proband was a 39-year-old Chinese male who claimed that he first began to experience night blindness 11 years ago. We revealed novel, missense compound heterozygous variants c. 2572G > A (p.V858I) and c. 2891G > A (p.R964Q) in the *CDH23* gene, which co-segregated with the disease phenotype causing Usher syndrome type ID (USH1D) in this Chinese pedigree. *CDH23* mRNA was highly expressed in the retina, and this protein was highly conserved as revealed by the comparison of *Homo sapiens* CDH23 with those from nine other species. This is the first study to identify the novel, missense compound heterozygous variants c. 2572G > A (p.V858I) and c.2891G > A (p.R964Q) of *CDH23*, which might cause USH1D in the studied Chinese family, thereby extending *CDH23* mutation spectra. Identifying *CDH23* pathogenic variants should help in the detailed phenotypic characterization of USH1D.

## Introduction

Usher syndrome is a group of genetically and clinically heterogeneous autosomal recessive diseases, such as progressive retinitis pigmentosa (RP) and sensorineural hearing loss (Boughman et al., 1983). Clinical differences have led to the categorization of three types of Usher syndrome: type I, II, and III (Keats and Corey, 1999; Mathur and Yang, 2015; Wei et al., 2018; Okano et al., 2019). Usher syndrome type I (USHI) is characterized by profound hearing impairment beginning at birth, vestibular dysfunction, and unintelligible speech in addition to RP (Okano et al., 2019).

Genetically, USHI is an autosomal recessive heterogeneous disorder causing mutations in at least eight genes that produces a similar disease or phenotype. These genes are: *MYO7A* (OMIM: 276903) at 11q13.5 causing USH1B (USH1A is considered to be the same; OMIM: 276900), *harmonin* or *USH1C* (OMIM: 605242) at 11p15.1 causing USH1C (OMIM: 276904), *CDH23* (OMIM: 605516) at 10q21-q22, causing USH1D (OMIM: 601067), *PCDH15* (OMIM: 605514) at 10q21.1 causing USH1F (OMIM: 602083), *SANS* or *USH1G* (OMIM: 607696) at 17q24-25 causing USH1G (OMIM: 606943), and *CIB2* (605564) at 15q24 causing USH1J (OMIM: 614869). Mutations in as yet unnamed genes in loci at 21q21 (USH1E; OMIM: 602097), 10p11.21-q21.1 (USH1K, OMIM: 614990), and 15q22-q23 (USH1H; OMIM: 612632) may also cause this type I phenotype. The clinical features of each are indistinguishable but are caused by different genes. Herein, Usher syndrome type ID (USH1D) is caused by homozygous or compound heterozygous mutation in the gene CDH23, a member of the cadherin superfamily which comprises calcium-dependent cell–cell adhesion glycoproteins. The *CDH23* gene locates on chromosome 10q22.1 and encodes a predicted protein of 3,354 amino acids with a single transmembrane domain and 27 cadherin repeats. During late embryonic or early postnatal development, the CDH23 protein was required for establishing and maintaining the proper organization of the stereocilia bundle of hair cells in the cochlea and the vestibule. However, the expression levels of *CDH23* in different tissues were not fully described.

The relationships between the variations in the Usher syndrome-caused genes and the resultant different Usher syndrome types/phenotypes in the patients are highly variable. Novel *CDH23* mutations in patients with USH1D and their relationships with genotypes/phenotypes are not well-documented in the Chinese population. Next-generation sequencing (NGS) provides a useful genetic diagnosis approach (Adams and Eng, 2018; Fu et al., 2019; Rahbaran et al., 2019). In the present study, novel missense compound heterozygous mutations of *CDH23* are revealed in Chinese pedigree links with USH1D.

## Case Presentation

**FIGURE 1:**
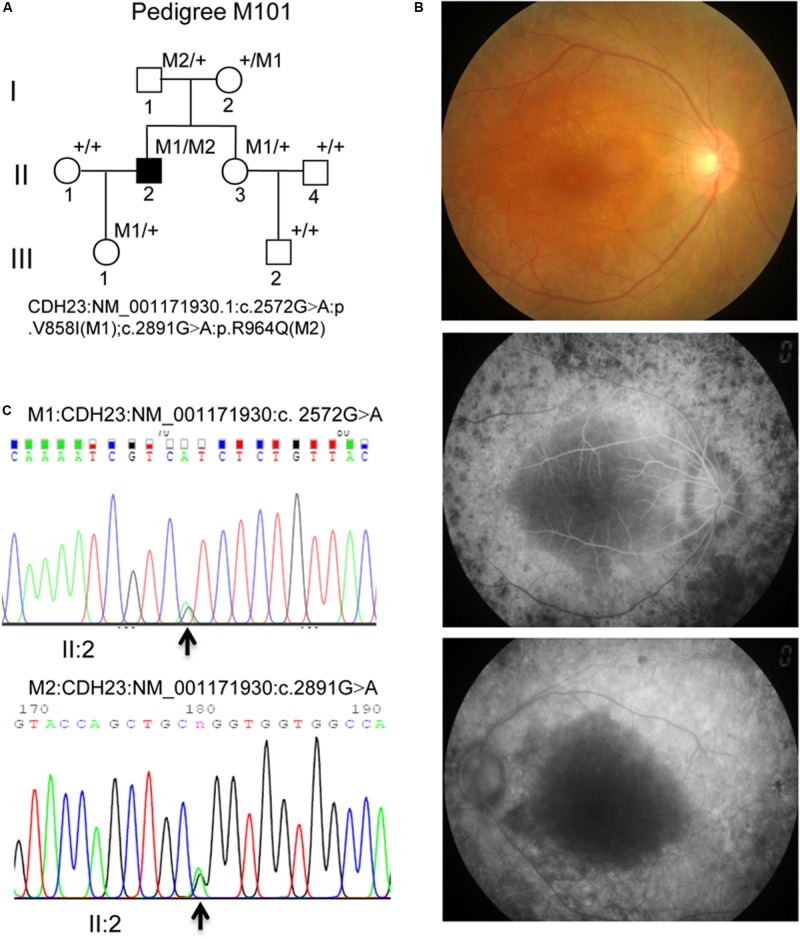
**(A)** M101 pedigree with Usher syndrome type ID. Normal individuals are shown as clear circles (females) or squares (male). The filled square indicates the proband (II: 2, arrow) with the compound heterozygous mutation of the *CDH23* gene: NM_001171930.1:exon22:c.2572G > A:p.V858I (M1); exon24: c. 2891G > A: p.R964Q(M2). “+,” wild-type allele. **(B)** Representative retinal phenotypes of proband II: 2. Top panel. Representatively FP in patient II: 2 of the right eye. Middle and bottom panel. Representatively FFA in patient II: 2 of both right and left eyes, respectively. **(C)** Pyrogram profiles for variant verification by Sanger sequencing, indicating the sequenced results in II: 2 (missense c. 2572G > A mutant type) (top panel), II: 2 (missense c.2891G > A mutant type) (bottom panel). The arrows show the mutation position NM_001171930.1: c.2572G > A or c. 2891G > A in the *CDH23* gene, respectively.

The M101 pedigree consisted of a proband and seven family members (Figure 1A). The proband (Figure 1, II: 2) was a 39-year-old Chinese male who claimed that he first began to experience night blindness 11 years ago. Fundus examinations showed macular changes in both eyes (Figure 1B). The retinal pigment epitheliums (RPEs) were atrophied (data not shown). The proband was first characterized as having RP and currently claims that they are presently experiencing hearing loss. Pure tone audiometry subsequently confirmed the presence of sensorineural hearing loss (data not shown). His parents and his daughter tested negatively for hearing loss. Therefore, the most likely diagnosis for the proband was Usher syndrome (type I).

The study has been approved by the Ethics Committee of Southwest Medical University/the Affiliated Huaian No. 1 People’s Hospital of Nanjing Medical University, and with the Helsinki Declaration of 1975, as revised in 2013. The protocol and procedures employed for the mouse experiments were ethically reviewed and approved by the Ethics Committee of Southwest Medical University/the Affiliated Huaian No. 1 People’s Hospital of Nanjing Medical University. The patient and the family members gave written informed consent to participate in the study, and for the publication of their clinical cases. All experimental works were carried out following the approved protocols and procedures.

## Methods

### Clinical Assessment, Blood Collection, and DNA Isolation

Clinical ophthalmic examinations and audiometric testing of the proband were performed as described previously (Wei et al., 2018; Huang et al., 2019). Blood samples from the family being studied were retrieved, and DNA was isolated from these samples. Healthy control blood samples in Luzhou were also collected (Fu et al., 2002; Cheng et al., 2019).

### Targeted NGS, Quality Control, and Bioinformatics Analysis

Targeted next-generation sequencing (TGS) analyses with 256 disease-causing genes panel were performed on an M101 DNA sample, according to the manual from Illumina as previously described (Zhang et al., 2016; Fu et al., 2017, 2018; Soens et al., 2017; An et al., 2019). Library construction, TGS, bioinformatic computational framework and data analysis were executed according to the manufacturer’s protocols (Koenekoop et al., 2012; Soens et al., 2016; Fu et al., 2017; Adams and Eng, 2018; An et al., 2019). Ninety-six percent of the targeted regions have coverage >20× and 91.1% of the targeted regions have coverage >40×. In all, more than 10 million bases of the sequence with 100-bp read length, 40,000 SNPs and 11,400 INDELs were generated. After quality assessment, more than 97% of billion bases were aligned to the human reference sequences (hg19) by BWA-MEM, duplicate reads were removed by Picard, local realignments were conducted by GATK, and variants were called by using Atlas2. A population frequency threshold of 0.5% was applied to filter out common variants which occur too frequently to be the cause of a rare Mendelian disease. Among those, billions of bases covered with a 10-fold coverage target region. Four NGS cohort databases were used to determine allele frequencies (Soens et al., 2016). The functional consequence of remaining rare variants was annotated using ANNOVAR, and dbNSFP was used to compile *in silico* predictions about the deleteriousness of non-synonymous variants. UGENE was used to perform the multiple sequence alignment using the MUSCLE alignment algorithm (Soens et al., 2016). The comprehensive and method of variant interpretation in detail has been described in An’s study (An et al., 2019).

The pathogenicity of each variant was assessed with the following databases: PolyPhen-2, Sorting Intolerant From Tolerant (SIFT), MutationTaster, and I-Mutant2.0 (Fu et al., 2018; Cheng et al., 2020). Variants, considered as pathogenic candidates, were searched for in the Human Gene Mutation Database (HGMD) and The Exome Aggregation Consortium (ExAC) to determine whether they had been reported previously.

### Sanger Sequencing

Primer pairs M101-CDH23-22 and M101-CDH23-24 were designed using the Primer3 program with genomic sequences containing NM_001171930.1: c.G2572A in exon 22 and c.G2891A in exon 24, respectively, in the *CDH23* gene (Supplementary Table 1). PCR amplification was conducted using gDNA as a template by above designed primer pairs. Purified the PCR products and then the products were used for Sanger sequencing (ABI-3500DX Genetic Analyzer, Thermo Fisher Scientific) using M101-CDH23-22L and M101-CDH23-24L, which are shown in Supplementary Table 1.

Co-segregation analysis of the M101 family was conducted based on Sanger sequencing results.

### Prediction of Protein Structure and Bioinformatics Analysis

The homologs of the *cdh23* gene (NM_001171930.1) in humans were analyzed as previously described. The conserved domains of CDD in CDH23 protein (NP_071407.4) were searched using the NCBI (Marchler-Bauer et al., 2017; Imani et al., 2018, 2019; Cheng et al., 2020).

### RNA-Sequencing and Reverse Transcription-PCR Profiles

The *CDH23* gene expression profiles were analyzed from RNA-sequencing (RNA-seq) data, which were retrieved from healthy human samples of 95 individuals representing 27 different tissues from the NCBI. The expression values of *CDH23* mRNA in different human tissues were retrieved from the NCBI.

RNA was isolated, and semi-quantitative RT-PCR was executed as previously reported (Fu et al., 2018). The primer pair for RT-cdh23 (RT-cdh23-nL and RT-cdh23-nR) targeted the mouse *Cdh23* gene and is listed in Supplementary Table 1 (Fu et al., 2018). The amplified PCR product was separated on 1.2% agarose gel three times (Cheng et al., 2019). The mouse β*-actin* gene served as a control.

## Results

### NGS Results and Co-segregation Analysis

Compound heterozygous variants (c. 2572G > A) at exons 22 and (c. 2891G > A) 24 in the *CDH23* gene (NM_001171930.1) with high confidence were identified in the proband, leading to the production of Isoleucine from Valine, and Glutamine from Arginine at amino acid positions 858 (V858I) and 964 (p.R964Q), respectively, in the CDH23 protein (NP_001165401) (Figure 1A, II: 2). Both variants c. 2572G > A (p.V858I) and c. 2891G > A (p.R964Q) were proven to be novel by searching ExAC and HGMD databases (Table 1).

**TABLE 1:** Characteristics of *CDH23* variants and the analysis of disease-causing effects.

		**Variation**								
**Gene**	**Exon**	Nucleotide*	Protein*	**Type**	**Status**	**Polyphen-2**	**Mutation Taster**	**I-Mutant2.0**	**SIFT**	**ExAC**
CDH23	22	c. 2572G > A	p.V858I	Missense	Heter	B(0.012)	DC (0.999)	DS	T(0.3)	Novel
	24	c. 2891G > A	p.R964Q	Missense	Heter	PD (0.98)	DC (0.999)	DS	T(0.31)	Novel

*CDH23*, cadherin related 23; c, variation at cDNA level; p, variation at protein level; Heter, heterozygote; B, benign; PD, probably damaging; DC, disease-causing; DS, decrease stability; T, tolerated; ExAC, Exome Aggregation Consortium. *All nucleotide and amino acid are abbreviated according to the International Union of Pure and Applied Chemistry (IUPAC).

The mutations c. 2572G > A (p.V858I) and c. 2891G > A (p.R964Q) in the *CDH23* gene were validated as compound heterozygous in the proband (Figure 1C; pedigree II: 1), with c. 2891G > A being inherited from his father (Supplementary Figure 1; pedigree I: 1) and c. 2572G > A being inherited from his mother (Supplementary Figure 1; pedigree I: 2), by Sanger sequencing. The proband’s daughter was found to be heterozygous c. 2891G > A with a normal phenotype (pedigree III: 1; Supplementary Figure 1), and the proband’s wife had a normal phenotype with alleles of wild-type (data not shown; pedigree II: 1). Therefore, it was ascertained that these variants in the *CDH23* gene in the proband and the variants were co-segregated with this clinical phenotype in the family members. Neither variant was identified in either 1000 Genomes or the ExAC Browser. Altogether, this finding presents co-segregation of the variants in this family and pinpoints possible roles in pathogenesis of USH1D.

### Functional Effects of the *CDH23* Mutations c.G2572A (p.V858I) and c.G2891A (p.R964Q) for USH1D Disease

**FIGURE 2:**
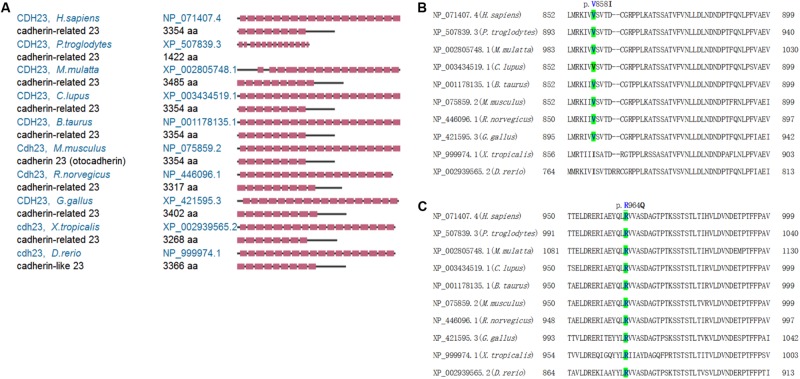
The CDH23 comparison and conserved mutant positions. **(A)** The conservation analysis of CDH23 in indicated species. **(B)** The conserved mutant position of p.V858 (highlighted in green). **(C)** The conserved mutant position of p.R964 (highlighted in green). Variants p.V858I and p.R964Q of CDH23 are indicated in Figures 3B,C, respectively.

The characteristics of *CDH23* mutations and the disease-causing effects on the proband are shown in Table 1. From Table 1, it can be observed that PolyPhen-2 analysis shows probable damage for p.R964Q change (score 0.98) and is benign for p.V858I change (score 0.012), respectively. MutationTaster revealed these changes to be caused by disease, with a score of 0.999. I-Mutant 2.0 indicated that both p.R964Q and p.V858I were damaged (score 0). Sorting Intolerant From Tolerant discovered tolerance for the p.R964Q change with a score of 0.31, and for the p.V858I change with a score of 0.30. Therefore, these missense mutations (c.2572G > A, p.V858I and c.2891G > A, p.R964Q) in the *CDH23* gene damage protein function resulting in a diagnosis of USH1D in the studied Chinese family based on the gene mutations and clinical phenotypes. By comparing *Homo sapiens* CDH23 protein to nine other species, including *Pan troglodytes*, *Macaca mulatta*, *Canis lupus*, *Bos taurus*, *Mus musculus*, *Rattus norvegicus*, *Gallus gallus*, *Xenopus tropicalis*, and *Danio rerio*, it was revealed that *CDH23* is a highly conserved ortholog (Figure 2A). Valine and Arginine at amino acid positions 858 and 964, respectively, were also found to be highly conserved (Figures 2B,C, highlighted in green). Taken together, our study reveals that the *CDH23* missense compound heterozygous variants c. 2572G > A (p.V858I) and c. 2891G > A (p.R964Q) might cause USH1D disease in the studied Chinese family.

### *CDH23* and *Cdh23* Expression Profiles

**FIGURE 3:**
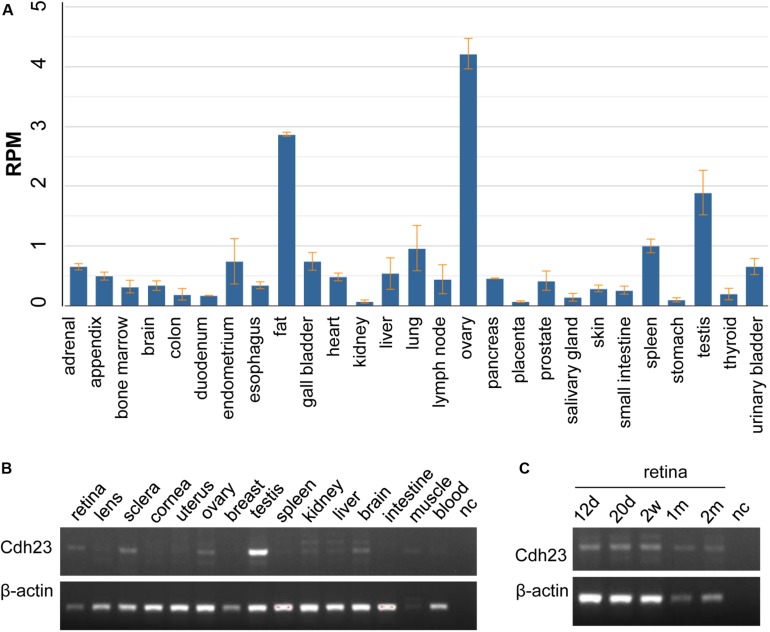
The mRNA expression in human *CDH23* and mouse Cdh23 tissues. **(A)** The *CDH23* mRNA expression in human tissues from RNA-seq data. **(B)** The *Cdh23* mRNA levels in the indicated tissues. **(C)** The *Cdh23* mRNA levels in the indicated development times in retinal tissue in mice. d, days; w, weeks; m, month(s); nc, no DNA template; muscle, skeletal muscle. Whole embryo eyeballs at 12.5 days (12 d) and 20.5 days (20 d) before birth, in panel **(C)**, respectively.

*CDH23* expression indicated that *CDH23* mRNA is highly expressed in the ovaries, with a value of RPKM 44.218 ± 0.225, followed by expression in fat and the testes, with the lowest expression in kidneys with a value of RPKM 0.072 ± 0.026 (Figure 3A and Supplementary Table 2). The expression values of RNA-seq in 27 different tissues are presented in Supplementary Table 2. No human eye tissues or developmental retinal stages were available. Therefore, *Cdh23* expression in mice was studied. From Figure 3B, it was found that *Cdh23* mRNA is highly expressed in the retina and testes, as well as in the sclera eye tissue and tissues of the ovaries, brain, and skeletal muscles. No or very low expression was identified in all other indicated tissues (Figure 3B). *Cdh23* is also expressed at the different developmental retinal stages and is highly expressed at the latter two stages (one and two months for the retina) (Figure 3C), indicating a role in retina maturation. Therefore, a vital role in the retina and other tissues for CDH23 was revealed through *CDH23* and *Cdh23* expression profiles.

## Discussion

Mutations in the *CDH23* gene cause USH1D (Bolz et al., 2001; Bork et al., 2001). Germline mutations in *CDH23* have also recently been identified and associated with both familial and sporadic pituitary adenomas (Zhang et al., 2017). The upregulation of this gene may also be associated with breast cancer. However, the relationships between the variations in the Usher syndrome-caused genes and the resultant different types of Usher syndrome diseases or phenotypes in patients are highly variable, and the genotype/phenotype correlations are divergent. Patients with mutations in less frequently mutated genes, including *CDH23*, have not been well-reported for Chinese patients with USH (Jiang et al., 2015; Sun et al., 2018). In the present study, by using TGS, Sanger sequencing, and co-segregation analysis, missense compound heterozygous pathogenic variants c. 2572G > A (p.V858I) and c. 2891G > A (p.R964Q) were successfully identified in the *CDH23* gene in a Chinese USH1D pedigree, thereby elucidating the genetic roles of the *CDH23* mutant alleles and confirming USH1D disease in this family. Therefore, *CDH23* mutations with USH1D and the relationship with the genotype/phenotype were successfully linked for the proband of this studied family. By searching the HGMD^1^ (access date, February 22, 2020), 275 pathogenic variants have been reported (Supplementary Table 3). Disease/phenotype and CDH23 mutation relationship was provided in Supplementary Table 4. To the best of our knowledge, the *CDH23* variants of c. 2572G > A (p.V858I) and c. 2891G > A (p.R964Q) are novel, thereby extending the *CDH23* mutation spectra.

By comparing *H. sapiens CDH23* to nine other species, it was revealed that the CDH23 protein is a highly conserved ortholog. *Cdh23* mRNA was only highly expressed in the retina, indicating its role in retinal functions. Taken together, this study revealed that the *CDH23* compound heterozygous variants c.2572G > A (p.V858I) and c.2891G > A (p.R964Q) cause disease linked to USH1D.

As a rare disease with common inherited forms resulting in combined visual and hearing impairment, up to 14 genes, including *MYO7A, CDH23, USH1C, PCDH15, USH1G*, and *CIB2* for USH type I, *USH2A, ADGRV1*, and *WHRN* for USH type II, *CLRN1* and *HARS* for USH type III, and *PDZD7*, *CEP250*, and *C2orf71*, are associated with Usher syndrome, with *CDH23* mutation identification in this study. Therefore, identifying *CDH23* pathogenic variants should help to fine map and characterize the clinical phenotype with USH1D.

Genetic screening and the accurate diagnosis of Usher syndrome in different populations is necessary (Ben-Rebeh et al., 2016; Sun et al., 2018; Santana et al., 2019). Efficient molecular diagnosis of Usher syndrome in a patient’s early childhood is of utmost importance, allowing better genetic counseling and therapeutic management (Ben-Rebeh et al., 2016; Ivanova et al., 2018; Maddalena et al., 2018; Jouret et al., 2019). With accurate gene diagnosis for Usher syndrome, the repair of specific mutations using a CRISPR/Cas9 editing system may be possible. Correction of the auditory phenotype in C57BL/6N mice via CRISPR/Cas9-mediated homology-directed repair in the mouse *Cdh23* gene, indicating that the CRISPR editing system has a potential for treating Usher syndrome (Mianne et al., 2016). Tauroursodeoxycholic acid, a taurine-conjugated bile acid that has been used in experimental research and for clinical applications in the liver, for diabetes, and neurodegenerative diseases, might prevent hearing loss and hair cell death in the study of Cdh23erl/erl mutant mice (Hu et al., 2016b). Salubrinal (Sal), the ER stress inhibitor, also delayed the progression of hearing loss and preserved hair cells in the Cdh23erl/erl mutant mice (Hu et al., 2016a). Therefore, the findings of this study assist in both understanding the molecular pathogenesis of USH1D and develop strategies for diagnosis, therapy, and genetic counseling for USH1D disease.

## Data Availability Statement

The datasets for this article are not publicly available due to participant/patient anonymity concerns. Requests to access the datasets should be directed to JF, fujunjiang@hotmail.com.

## Ethics Statement

The protocol and procedures employed for mouse were ethically reviewed and approved by the Ethics Committee of Southwest Medical University/the Affiliated Huaian No. 1 People’s Hospital of Nanjing Medical University. The study has been approved by the Ethics Committee of Southwest Medical University/the Affiliated Huaian No. 1 People’s Hospital of Nanjing Medical University, and with the Helsinki Declaration of 1975, as revised in 2013. All three patients gave written informed consent agreeing to participate in the study and for the publication of their clinical cases.

## Author Contributions

JuF, HL, and SS oversaw the project design and concept of the study. LZ, JiF, and JC performed DNA extraction, PCR amplification, sequencing, and data analysis. HL, QZ, and CD recruited the clinical patients and oversaw the clinical assessments. MK revised the manuscript. JuF wrote and revised the manuscript.

## Conflict of Interest

The authors declare that the research was conducted in the absence of any commercial or financial relationships that could be construed as a potential conflict of interest.

## Footnotes

1 http://www.hgmd.cf.ac.uk/ac/gene.php?gene=CDH23
