# Supplementary material for: Targeted Next-Generation Sequencing Identified Novel Compound Heterozygous Variants in the CDH23 Gene Causing Usher Syndrome Type ID in a Chinese Patient
Source: Front Genet. 2020 Apr 30;11:422. doi: 10.3389/fgene.2020.00422 (PMC7204213; doi:10.3389/fgene.2020.00422)

**Supplementary Material**

| **Supplementary Table 1: The sequences of PCR primers and PCR product sizes** | | | | |  |  |
| --- | --- | --- | --- | --- | --- | --- |
| Primer name | Left primer | Sequence (5′-3′) | Right primer | Sequence (5′-3′) | Size | °C |
| M101-CDH23-22 | M101-CDH23-22L | ctacagcctcaacagcacca | M101-CDH23-22R | gagagggtggcagattcaga | 375 | 60 |
| M101-CDH23-24 | M101-CDH23-24L | acttcttgggggagaagcat | M101-CDH23-24R | gagcctgtgcctacaagtcc | 413 | 60 |
| RT-cdh23 | RT-cdh23-nL | ctgacagtgaagggcactga | RT-cdh23-nR | gtggtcgggtacactctcgt | 387 | 60 |
| RT-b-actin-m | RT-b-actin-mL | tgttaccaactgggacgaca | RT-b-actin-mL | tctcagctgtggtggtgaag | 392 | 60 |

| **Supplementary Table 2. Expression values of CDH23 mRNA in human different tissues** | | | |
| --- | --- | --- | --- |
| Sample | Numbers | RPKM values | Counts |
| adrenal | 3 | 0.653 ± 0.053 | 122444 |
| appendix | 3 | 0.498 ± 0.067 | 83784 |
| bone marrow | 4 | 0.322 ± 0.106 | 143469 |
| brain | 3 | 0.338 ± 0.079 | 69675 |
| colon | 5 | 0.193 ± 0.096 | 90421 |
| duodenum | 2 | 0.169 ± 0.003 | 19095 |
| endometrium | 3 | 0.744 ± 0.379 | 142900 |
| esophagus | 3 | 0.344 ± 0.057 | 104667 |
| fat | 3 | 2.871 ± 0.032 | 538554 |
| gall bladder | 3 | 0.743 ± 0.148 | 232251 |
| heart | 4 | 0.483 ± 0.065 | 206938 |
| kidney | 4 | 0.072 ± 0.026 | 14919 |
| liver | 3 | 0.539 ± 0.264 | 102122 |
| lung | 5 | 0.965 ± 0.379 | 320788 |
| lymph node | 5 | 0.445 ± 0.243 | 223545 |
| ovary | 2 | 4.218 ± 0.255 | 1024253 |
| pancreas | 2 | 0.458 ± 0.007 | 103928 |
| placenta | 4 | 0.071 ± 0.012 | 30711 |
| prostate | 4 | 0.421 ± 0.161 | 106303 |
| salivary gland | 3 | 0.142 ± 0.064 | 55375 |
| skin | 3 | 0.29 ± 0.056 | 95299 |
| small intestine | 4 | 0.261 ± 0.067 | 69618 |
| spleen | 4 | 1.002 ± 0.114 | 348391 |
| stomach | 3 | 0.1 ± 0.035 | 24399 |
| testis | 7 | 1.894 ± 0.372 | 1511503 |
| thyroid | 4 | 0.196 ± 0.096 | 83791 |
| urinary bladder | 2 | 0.656 ± 0.135 | 132066 |

| **Supplementary Table 3.Other reported CDH23 mutations** | |
| --- | --- |
| Mutation type | Number of mutations |
| Missense/nonsense | 182 |
| Splicing | 38 |
| Small deletions | 38 |
| Small insertions | 8 |
| Small indels | 3 |
| Gross deletions | 2 |
| Gross insertions/duplications | 4 |

| **Supplementary Table 4. Disease/phenotype and CDH23 mutation numbers** | |
| --- | --- |
| Disease/phenotype | Number of mutations |
| Usher syndrome 1 | 64 |
| Usher syndrome 1d | 60 |
| Non-syndromic autosomal recessive deafness | 28 |
| Hearing loss | 24 |
| Hearing loss, non-syndromic | 14 |
| Hearing loss, non-syndromic ? | 14 |
| Deafness, nonsyndromic | 11 |
| Hearing loss, autosomal recessive ? | 8 |
| Non-syndromic autosomal recessive deafness ? | 6 |
| Deafness, autosomal recessive 12 | 5 |
| Deafness, non-syndromic, autosomal recessive | 5 |
| Usher syndrome | 5 |
| Deafness, non-syndromic | 4 |
| Usher syndrome 1 ? | 4 |
| Usher syndrome 2 | 4 |
| Deafness | 3 |
| Deafness ? | 3 |
| Usher syndrome 1d ? | 3 |
| Hearing loss ? | 2 |
| Sector retinitis pigmentosa & hearing loss | 2 |
| Prelingual hearing loss | 1 |
| Sensorineural hearing loss | 1 |
| Sensorineural hearing loss, nonsyndromic ? | 1 |
| Usher syndrome ? | 1 |
| Usher syndrome 1, modifier of ? | 1 |
| Usher syndrome 2 ? | 1 |

**Supplementary Figure 1**


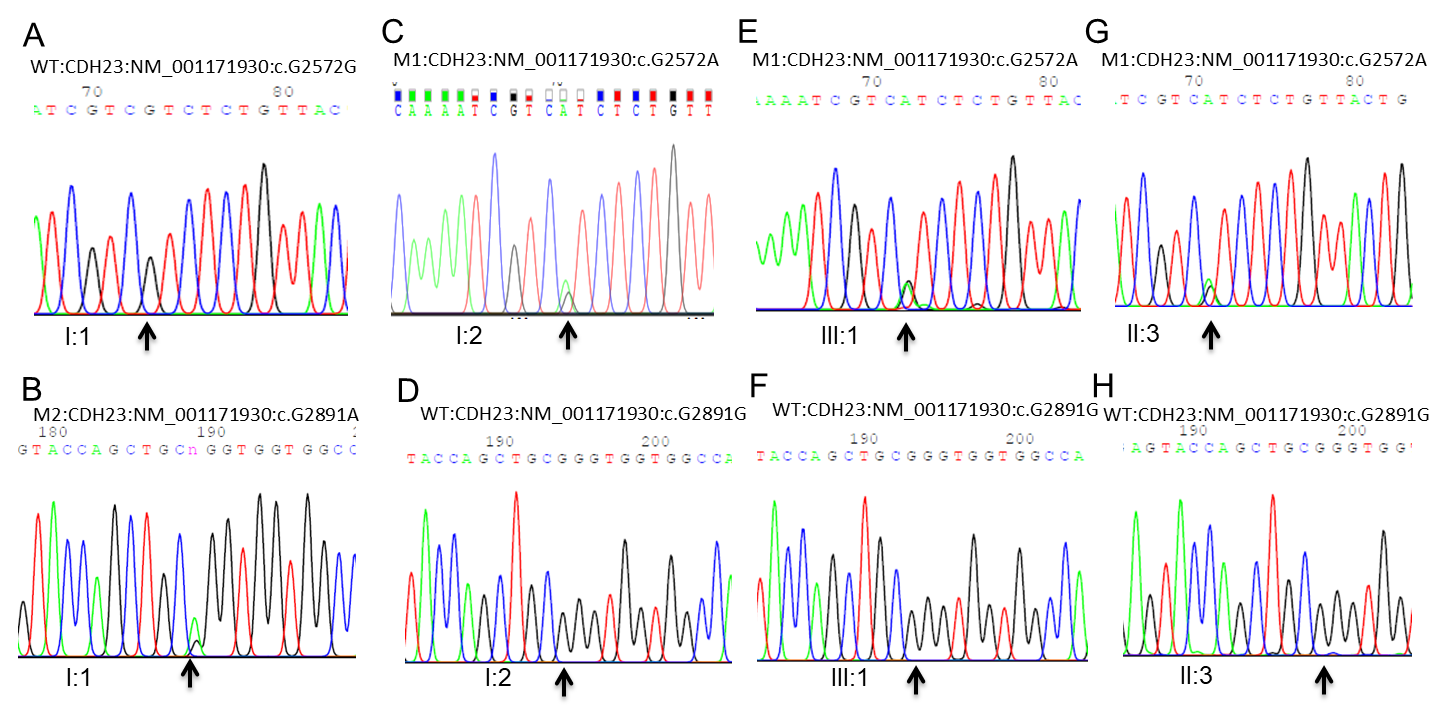

Supplement: Supplementary file 1 [file Data_Sheet_1.doc]
